# A report of a pedigree with compound heterozygous mutations in the *SLC22A5* gene

**DOI:** 10.3389/fped.2023.985720

**Published:** 2023-06-06

**Authors:** Yunguo Zhou, Yucai Liu, Yang Shen, Fang Xu, Fei Xu, Hui Huang, Junkai Duan

**Affiliations:** ^1^ Jiangxi Provincial Children's Hospital, Nanchang, China; ^2^ JXHC Key Laboratory of Children's Cardiovascular Diseases, Jiangxi Provincial Children's Hospital, Nanchang, China; ^3^ Medical College of Nanchang University, Nanchang, China; ^4^ Department of Genetic Medicine, Jiangxi Key Laboratory of Molecular Medicine, The Second Affiliated Hospital of Nanchang University, Nanchang, China

**Keywords:** primary carnitine deficiency, SLC22A5 gene, cardiomyopathy, gene, pediatrics

## Abstract

**Introduction:**

To investigate the clinical characteristics and disease outcomes of a pedigree with compound heterozygous mutations in the *SLC22A5* gene.

**Methods:**

Serum acylcarnitine profiles of patients were analyzed using tandem mass spectrometry. DNA samples isolated from patients and their first-degree relatives were subjected to high-throughput sequencing, and mutations were validated using Sanger sequencing.

**Results:**

The proband, a 4-month-old girl, presented with seizure episodes and mild cardiac hypertrophy and was diagnosed with primary carnitine deficiency (PCD), with carnitine levels of 5.165 mol/L. Her brother, a 6-year-and 4-month-old boy, was also diagnosed with PCD with serum-free carnitine levels of 1.014 mol/L (reference values 10–60 mol/L). Compound heterozygous mutations (c.760C > T [p.R254X] and c.825G > A [p.W275X]) were detected in the *SLC22A5* gene in both patients and were inherited from the mother and father, respectively. Oral L-carnitine significantly improved or resolved the clinical symptoms.

**Conclusion:**

Children with compound mutations in *SLC22A5* may present different clinical manifestations, particularly at different ages. Early clinical manifestations have a greater impact on the organs and may cause irreversible damage. PCD can cause epilepsy and dilated cardiomyopathy. Tandem mass spectrometry and high-throughput sequencing are recommended to confirm the diagnosis. Early L-carnitine supplementation can improve symptoms and reverse organ damage in some children. Tandem mass spectrometry should be used to screen for newborns with a family history of PCD.

## 1. Introduction

Primary carnitine deficiency (PCD), also known as primary carnitine uptake defect (CUD) or carnitine transporter deficiency (CTD), is caused by defective organic cation transporter 2 (OCTN2), which is an organic cation/carnitine transporter encoded by *SLC22A5*; mutations in *SLC22A5* result in increased urinary excretion of carnitine; carnitine deficiency in blood, tissues, and cells; and defective fatty acid oxidation. PCD is an autosomal recessive genetic disease (1). Typical manifestations include metabolic disorders during infancy, cardiomyopathy during childhood, and fatigue during adulthood. Patients with PCD may also be asymptomatic (2). Early diagnosis and long-term treatment are essential to achieve optimal clinical outcomes. Newborn PCD screening is routinely performed in several countries. This study retrospectively analyzed the clinical and follow-up data of a pedigree of patients with PCD treated at the Pediatric Heart Disease Treatment Center of Jiangxi Provincial Children's Hospital in February 2021.

## 2. Clinical data

### 2.1. Case 1

**Figure 1:**
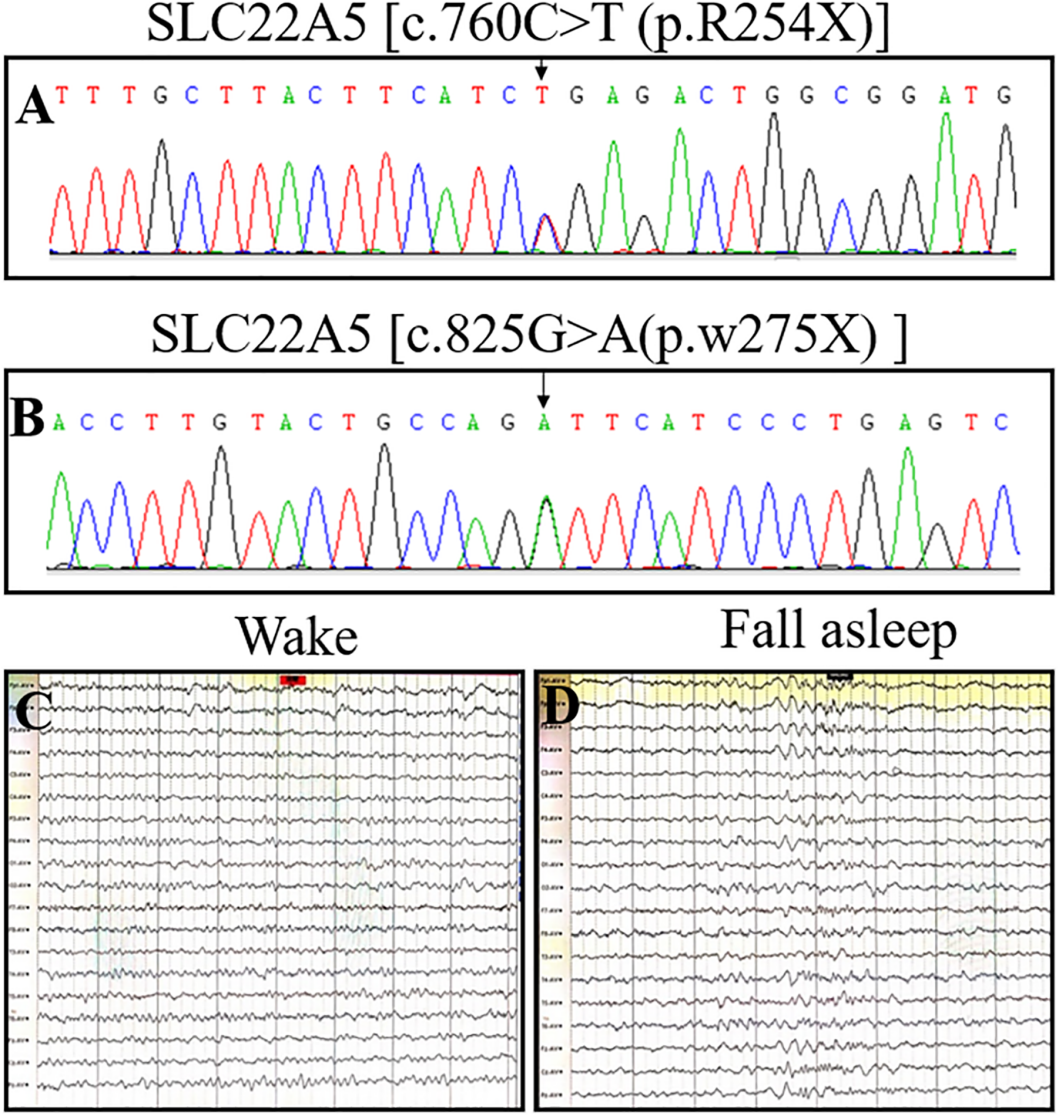
OCTN2 mutations and electroencephalograms of a 4-month-old girl (the proband) with primary carnitine deficiency. (**A**) Mutation [c.760C > T (p.R254X)] in the *SLC22A5* gene in the proband. (**B**) Mutation [c.825G > A (p.w275X) ] in the *SLC22A5* gene in the proband. (**C** and **D**) EEG of the proband was recorded in November 2021 when she was 4 years and 8 months old and had already received four years of treatment. The electroencephalogram indicated no seizures on either wakefulness or Non-REM Phase II.

The proband, a 4-month-old girl, was delivered *via* cesarean section at gestational week 40. Her perinatal period was unremarkable, with birth weight and height of 3.12 kg and 50 cm, respectively. At four months of age, she was unable to hold her head in a stable position. In July 2017, she was admitted to the Children's Hospital of Chongqing Medical University (CHCMU) for diarrhea and vomiting for a day, lethargy for half a day, and convulsions. After admission, she experienced one seizure episode, manifesting as eye gazing and upper limb rigidity for minutes, followed by drowsiness. The results of the physical examination were as follows: temperature, 37.6°C; light coma; slow bilateral pupillary light reflex; heart rate, 130 bpm; liver extending to 3 cm below the right rib cage; spleen extending to 1 cm below the rib cage; left Babinski's sign (+, weak); right Babinski's sign (+); deep and superficial reflexes (+); and capillary refill time, 3 s. The laboratory test results were as follows: alanine aminotransferase (ALT), 302 U/L; aspartate aminotransferase (AST), 607.7 U/L; blood ammonia, 111.6 µmol/L; and procalcitonin, 1.034 ng/mL. Blood gas analysis results were as follows: pH, 7.13; CO_2_ pressure (pCO_2_), 12 mmHg; O_2_ pressure (pO_2_), 119 mmHg; and blood base excess (BE), 22.8 mmol/L. Urine analysis results indicated the presence of ketone bodies (++), whereas stool analysis results indicated white blood cell (WBC) (++) and rotavirus (+). Blood cultures were positive for *Staphylococcus epidermidis* (+). Chest radiography revealed bilateral pneumonia. Electroencephalogram (EEG) showed increased 1.5 − 3.5 Hz *δ* waves under a light coma, with two subclinical seizure episodes. Echocardiography revealed slight thickening of the ventricular septum and posterior wall of the left ventricle, mild tricuspid regurgitation, and normal left ventricular systolic function. The patient received normal saline for volume expansion, mannitol to lower intracranial pressure, reduced glutathione to protect liver function, dibutyryl cycloadenosine monophosphate to nourish the cardiac muscle, and vitamin B6 to stop the spasms. On the same day, the patient experienced sudden irritability, face and lip cyanosis, and breathing difficulties. The patient was administered inhaled oxygen, anti-infective therapy, creatine phosphate, and L-carnitine (500 mg/day) to nourish the cardiac muscle; oxiracetam to protect brain function; and symptomatic care to restore homeostasis. The patient's condition improved after the symptomatic treatment. Blood bacterial culture was negative, liver function improved, and the WBC count was normal. She experienced convulsions (1–8 episodes/day). Oral levetiracetam solution (32.5 mg, daily) was administered as anti-epileptic therapy. Brain magnetic resonance imaging (MRI) revealed slightly widened extracerebral spaces around the frontal, temporal, and parietal lobes on both sides (up to 5 mm), with a small amount of subdural effusion. Tandem mass spectrometry revealed significantly elevated levels of serum-free carnitine and various acylcarnitines, potentially related to the use of L-carnitine. High-throughput sequencing of inherited metabolic disorders and mitochondrial diseases revealed compound heterozygous mutations in *SLC22A5* (Figures 1A,B; Table 1), including c.760C > T (p.R254X) and c.825G > A (p.w275X), inherited from the mother and father, respectively. The parents of the patient are heterozygous carriers. Fourteen days after treatment, the patient was conscious and had no fever, cough, wheezing, or shortness of breath. Her seizures improved (1–3 episodes/week), and her condition remained stable. After discharge, the patient experienced significantly more frequent seizures because her parents reduced the L-carnitine dose without consultation. Tandem mass spectrometry revealed free carnitine deficiency (5.165 mol/L). The dose of the oral L-carnitine solution was increased to 1,500 mg, which reduced the number of seizures. Repeat tandem mass spectrometry showed a normal free carnitine concentration, and the frequency of seizures was 1–5 episodes/month. In November 2021, the proband underwent a follow-up EEG (Figures 1C,D), and brain MRI results were normal; subsequently, the dose of levetiracetam was adjusted. At present, the patient is consuming 1,500 mg of levocarnitine (approximately 100 mg/kg), 250 mg of levetiracetam (approximately 21 mg/kg), and 75 mg of oxcarbazepine (approximately 6.25 mg/kg). She experiences 1–2 seizures every 3–4 months. The development of the patient is slower than those of normal children of the same age. She was able to hold her head in a stable position by 10 months of age, walk by 2 years, and talk by 2.5 years of age.

**Table 1:** Sequencing results (proband).

Gene	Locus	Test	Nucleotide change	Subject	Result
*SLC22A5*	chr5-131721127	Sanger sequencing	c.760C > T	Proband	Heterozygous mutation
*SLC22A5*	chr5-131722717	Sanger sequencing	c.825G > A	Proband	Heterozygous mutation

### 2.2. Case 2

**Figure 2:**
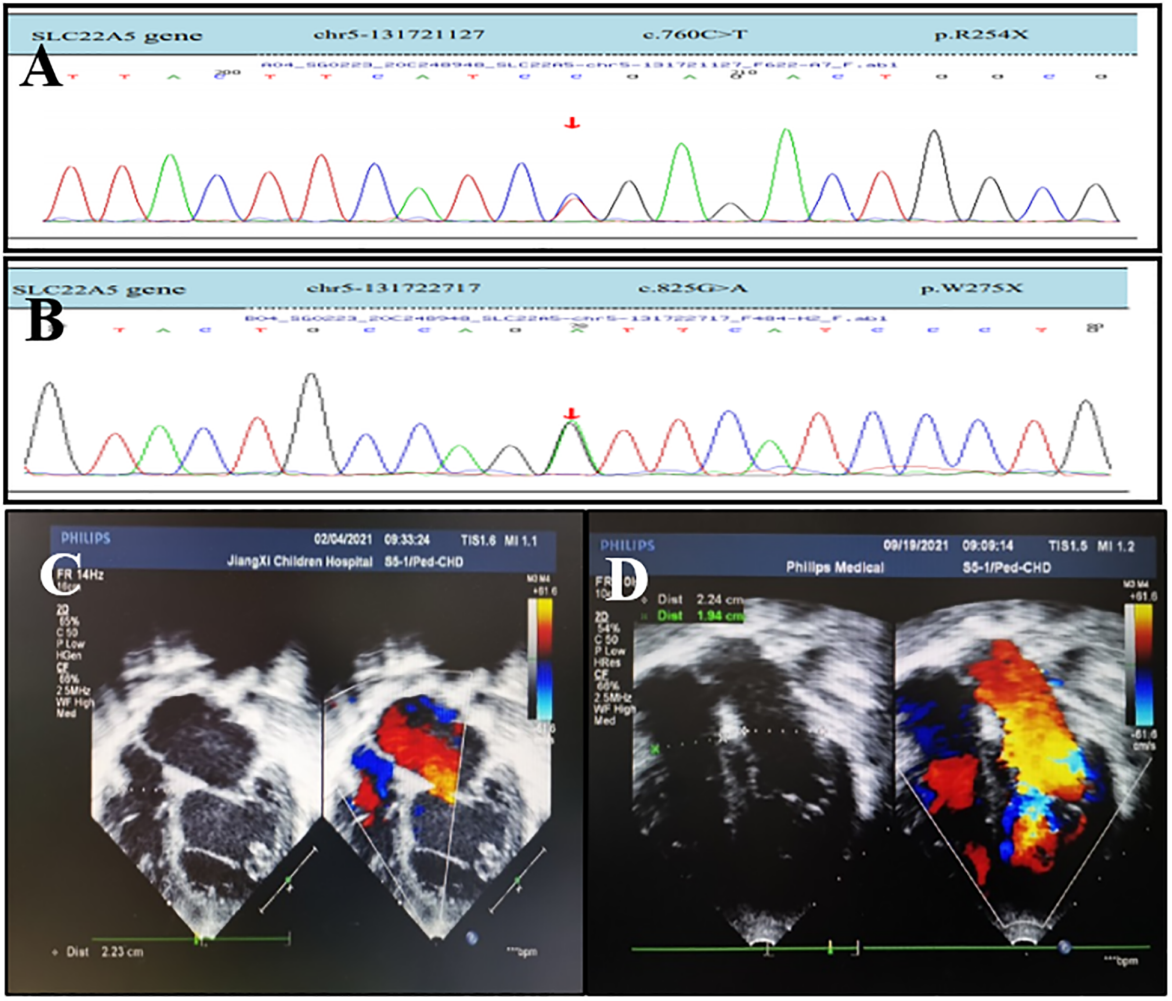
OCTN2 mutations and echocardiography of a 6-year-and 4-month-old boy (the proband's brother) with primary carnitine deficiency. (**A**) Mutation [c.760C > T (p.R254X)] in the *SLC22A5* gene. (**B**) Mutation [c.825G > A (p.w275X) ] in the *SLC22A5* gene. The arrows indicate the site of the mutation. (**C**) Transthoracic echocardiography (TTE) of the boy was performed in February 2021 when he was 6 years and 4 months old and did not receive treatment. (**D**) TTE of the boy after 6 months of treatment in September 2021. TTE parasternal four-chamber view shows diameter changes of heart chambers before and after 6 months of treatment.

The older brother of the proband, a boy aged 6 years and 4 months was delivered at full term. His perinatal period was normal with a birth weight of 4.3 kg. His growth and development were lower than those of normal children of the same age. In February 2021, he was admitted to the Department of Gastroenterology of our hospital for recurrent abdominal pain for more than one month. Physical examination revealed lethargy, dull paroxysmal pain below the xiphoid process, tenderness (no radiating pain), vomiting of stomach contents, no fever or cough, no convulsions, chest tightness, chest pain, diarrhea, or abdominal distension. Laboratory tests showed an NT-ProBNP concentration of 5,206.6 pg/mL and normal liver and kidney functions, blood test results, and electrolyte levels. Brain MRI results were unremarkable. Chest radiography (in a standing position) revealed cardiac enlargement with a cardiothoracic ratio of approximately 0.73. Echocardiography revealed a significantly enlarged left ventricle, left ventricular systolic dysfunction, and severe mitral regurgitation (left ventricular end-diastolic diameter (LVEDd), 71 mm; left ventricular end-systolic diameter (LVESd), 59 mm; left atrial diameter (LA), 32 mm; left ventricular ejection fraction (LVEF), 34%). Tandem mass spectrometry showed decreased levels of serum-free carnitine (1.014 mol/L; reference: 10–60 μmol/L) and various acylcarnitines (e.g., acetylcarnitine, butyrylcarnitine, and lauroylcarnitine), suggesting PCD. The patient received levocarnitine 2,000 mg IV (117 mg/kg/day), oral captopril, digoxin to strengthen cardiac function, and furosemide and spironolactone (diuresis) for 7 days. Tandem mass spectrometry showed that the levels of serum-free carnitine and various acylcarnitines returned to normal (41.426 μmol/L), and echocardiography suggested steady improvement. The treatment was switched to oral levocarnitine (3,000 mg, 176 mg/kg). After 17 days of treatment, the abdominal pain dissipated and his appetite improved. Echocardiography revealed the following findings: LVEDd, 41 mm; LVESd, 29 mm; LA, 27 mm; and LVEF, 59%. Six months later, echocardiography results were as follows: LVEDd, 38 mm; LVESd, 21 mm; LA, 22 mm; and LVEF, 61% (Figures 2C,D; Table 2). Laboratory tests suggested that the NT-proBNP concentration had returned to normal levels (68 pg/mL; reference: 0–125 pg/mL). After treatment, the diameters of the enlarged left atrium and left ventricle decreased to normal, and the LVEF increased to normal values.

**Table 2:** Echocardiography before and after L-carnitine treatment.

	Before treatment	After 4 days of treatment	After 14 days of treatment	After 17 days of treatment	After 47 days of treatment	After 116 days of treatment	After 210 days of treatment
LVDd (mm)	71	62	46	41	40	38	38
LVDS (mm)	59	21	35	29	28	21	21
LA (mm)	32	24	25	27	20	16	22
LVEF (%)	34	36	48	59	56	76	61

LVEDd, left ventricular end-diastolic diameter; LVEDs, left ventricular end-systolic diameter; LA, left atrial diameter; LVEF, left ventricular ejection fraction.

High-throughput sequencing was used to screen for mutations. Bioinformatics and clinical information analytics were used to analyze genetic data, and Sanger sequencing was used to confirm *SLC22A5* mutations in the patient and his first-degree relatives. The results indicated compound heterozygous mutations in *SLC22A5*, including c.760C > T (p.R254X) and c.825G > A (p.w275X), inherited from the mother and father, respectively (Figures 2A,B; Table 3). Both parents are heterozygous carriers. The patient harbored the same mutations as the proband. Echocardiography and serum tandem mass spectrometry results were normal in the parents.

**Table 3:** Sequencing results for the proband's older brother.

Gene	Locus	Test	Nucleotide change	Subject	Result
*SLC22A5*	chr5-131721127	Sanger sequencing	c.760C > T	Older brother of the proband	Heterozygous mutation
*SLC22A5*	chr5-131722717	Sanger sequencing	c.825G > A	Older brother of the proband	Heterozygous mutation

## 3. Discussion

PCD is a result of defective OCTN2 caused by *SLC22A5* mutations; this results in increased urine excretion of carnitine; carnitine deficiency in blood, tissues, and cells; and defective fatty acid oxidation (3). PCD occurs in one in 300 to 120,000 individuals (4). In the US, the incidence is 1 per 20,000–70,000 individuals (3), while in China, the incidence is 1 per 45,000 individuals (5). Most clinical manifestations of PCD are nonspecific and vary significantly with the age of onset, organ involvement, and severity. Common symptoms in infants include acute metabolic disorders, manifesting as feeding problems, vomiting, confusion, hepatomegaly, hypoketotic hypoglycemia, elevated liver enzymes, and hyperammonemia (6). In young children, a common symptom is a dilated or hypertrophic cardiomyopathy, especially dilated cardiomyopathy, with an average age of onset of 2–4 years (7). Most adult patients are asymptomatic or have mild symptoms that manifest as decreased endurance and susceptibility to fatigue (8). Atypical clinical manifestations include recurrent nausea, abdominal pain, anemia, proximal muscle weakness, developmental delay, respiratory distress, intellectual and motor disabilities, mental and behavioral disorders, autism spectrum disorders, epilepsy, and susceptibility to infection. Most carriers are asymptomatic (9, 10). A few reports have observed sudden cardiac death in seemingly asymptomatic adult patients with PCD in the Faroese population, necessitating the treatment of all diagnosed individuals and maintaining L-carnitine supplementation regardless of the presence or absence of symptoms (11, 12).

The brother and sister of the family, the cases of which are presented in this retrospective study, both had PCD, with identical mutations, but their disease characteristics were very different. The proband mainly had systemic symptoms caused by a typical metabolic disorder that manifests as diarrhea, vomiting, poor spirit, convulsions, and repeated seizures. Liver function, urine organic acids, chest x-ray, EEG, and brain MRI showed different levels of abnormalities, and the cardiac abnormalities were mild. The older brother of the proband showed cardiac insufficiency but no metabolic disorders or convulsions. Abnormal liver function and an increased cardiothoracic ratio were secondary changes in cardiac insufficiency, and they all normalized after supplementary treatment with carnitine. Studies have shown that Carnitine deficiency was found in approximately 17% of patients with epilepsy (13). Yang et al. (10) also reported a case of PCD with epilepsy. In the proband's older brother, the initial symptom was abdominal pain with an ongoing poor appetite and decreased endurance. Echocardiography revealed a dilated cardiomyopathy. Studies have shown that R245X and V295X mutations in *SLC22A5* may be associated with cardiomyopathy, which may be the only clinical phenotype (14).

Tandem mass spectrometry is often used in clinically suspected patients with PCD to measure the levels of serum-free carnitine (C0) and various acylcarnitines (cut-off value: serum C0 < 10 µmol/L or below the self-defined lower limit) (14). Using this method, serum C0 was found to be low in both children in this study. For the proband, the initial free carnitine level was relatively high because she consumed L-carnitine before tandem mass spectrometry; however, she was found to have a free carnitine deficiency in a repeat test because she did not consume L-carnitine as instructed after discharge. Genetic sequencing detected *SLC22A5* mutations, confirming the presence of PCD.

Studies have shown that the most common mutation associated with PCD in China is c.760C > T (p.R254X), which accounts for approximately 25.6% of all cases (5). However, Huang et al. found that the proportion of mutation sites in newborns and mothers with PCD is not the same, and the genetic analysis of *SLC22A5* showed that p.S467C, p.F17l, and p.R254X were the three most common variants in newborns with PCD. In mothers with PCD and healthy children, p.S467C, p.F17l, and R399W were the three most common variants, whereas the severe variant p.R254X was rare, indicating that the current screening program may fail to detect all newborns with PCD and underestimate the incidence rate of PCD (15). Homozygous and compound heterozygous mutations can cause PCD. The severity of the disease varies depending on the mutation site; p.R254X is a severe variant in newborns but is rare in adults and healthy children. Possibly, patients harboring the p.R254X variant may not survive in childhood or may not have entered the delivery population due to illness, and hence cases of the p.R254X variant are rare. In this report, both patients had compound heterozygous mutations in *SLC22A5*, including c.760C > T (p.R254X) and c.825G > A (p.w275X); however, the disease onset age and symptoms were different, indicating clinical heterogeneity, which may be due to other genetic changes and interactions among non-genetic factors. The risk of developing PCD, an autosomal recessive disease, is 25% per pregnancy in heterozygous parents. Moreover, 0.5 − 1% of the general population are heterozygous carriers.

In this study, both parents of the proband were carriers. After the proband showed clinical symptoms, her older brother did not undergo PCD screening, excluding early L-carnitine treatment. He eventually showed clinical symptoms when he was 6 years and 4 months old. These data indicate that PCD screening and targeted *SLC22A5* sequencing should be performed for all family members once the first PCD case has been identified. Moreover, individuals with *SLC22A5* mutations should consider chorionic villus sampling or amniocentesis, which could aid in prenatal diagnosis (1, 3). Carnitine transports long-chain fatty acids from the cytoplasm to the mitochondrial matrix to enable *β*-oxidation and is also involved in the transfer of peroxisomal-oxidation products. Other functions of carnitine include the regulation of the acyl-coenzyme A/coenzyme A ratio, energy storage in the form of acetylcarnitine, and regulation of the toxic effects of poorly metabolized acyl groups by binding and excreting urinary carnitine esters (2). When serum carnitine levels are 10 − 20% below normal, carnitine deficiency may cause clinical symptoms in the liver, heart, muscles, and brain (16). L-carnitine supplementation is the main treatment for PCD. For severe acute cases, the initial dose is 100–400 mg/kg/day, with three doses administered over a day (oral or intravenous). The dose should be adjusted based on serum C0 to maintain normal acylcarnitine levels and C0 of ≥20 mmol/L (13). Patients with PCD must receive L-carnitine therapy throughout their life. Sudden withdrawal can lead to a sharp decrease in serum carnitine levels and may result in mortality (10). Studies have shown that the lack of treatment increases the risk of sudden cardiac death and cardiac scarring. Sudden cardiac death may occur in patients with PCD treated with L-carnitine (17). PCD-induced cardiomyopathy is treatable and may recover completely with early treatment (7). In this report, the LVEF, LVEDd, LVESd, and LA returned to normal 15 days after L-carnitine treatment in the proband's older brother.

Jun reported that it was difficult to reverse intellectual and motor disabilities in a 3-year-old child with PCD and hypoglycemic encephalopathy (18). In this report, the proband continued to have seizures and developmental delays, suggesting that early onset has a greater impact on organs and may even cause irreversible damage. Although brain MRI and EEG findings were normal during 4-year and 4 month follow-up, her brain function was irreversibly damaged. The patient was consuming levocarnitine, levetiracetam, and oxcarbazepine; however, she still experienced seizures every 3–4 months. At present, it is unclear whether the seizures will disappear or whether intellectual and motor disabilities will be reversed. Some studies have shown that seizures can be completely controlled after surgical resection of structural brain abnormalities (10). However, treatment with carnitine was recommended as the first line of treatment. Carnitine supplementation should be initiated as soon as possible before irreversible organ damage occurs. Carnitine supplementation improves metabolic decompensation and skeletal and myocardial functions. If individuals continue to receive carnitine supplementation, their long-term prognosis is favorable (19).

## Data Availability

The datasets presented in this study can be found in online repositories. The names of the repository/repositories and accession number(s) can be found below: https://www.ncbi.nlm.nih.gov/, 603377.
